# The Ottawa score for prediction of recurrent venous thromboembolism in cancer patients treated with tinzaparin: an individual patient data meta-analysis

**DOI:** 10.1016/j.rpth.2025.103278

**Published:** 2025-12-02

**Authors:** Céline Chapelle, Philippe Girard, Luis Jara-Palomares, Agnès Y.Y. Lee, Olivier Sanchez, Guy Meyer, Géraldine Poenou, Patrick Mismetti, Isabelle Mahé, Silvy Laporte

**Affiliations:** 1 Jean Monnet University, Mines Saint-Etienne, INSERM, U1059, SAINBIOSE, F-42023, Saint-Etienne, France; 2 Service de Pharmacologie Clinique, CHU Saint-Etienne, F-42055, Saint-Etienne, France; 3 F-CRIN INNOVTE Network, Saint-Etienne, France; 4 Département de Pneumologie, Institut Mutualiste Montsouris, Paris, France; 5 Respiratory Department, Medical Surgical Unit of Respiratory Diseases, Hospital Virgen del Rocio, Seville, Spain; 6 Center for Biomedical Research in the Respiratory Diseases Network (CIBERES), Instituto de Salud Carlos III, Madrid, Spain; 7 University of British Columbia, Vancouver, British Columbia, Canada; 8 BC Cancer, Vancouver, British Columbia, Canada; 9 Université Paris Cité, Paris, France; 10 Service de Pneumologie et Soins Intensifs, Hôpital Européen Georges Pompidou, APHP, Paris, France; 11 INSERM UMR_S970, Cardiovascular Research Center, Team “Endotheliopathy and Hemostasis Disorders”, Paris, France; 12 Service de Médecine Vasculaire et Thérapeutique, CHU Saint-Etienne, Hôpital Nord, Saint-Etienne, France; 13 Assistance Publique-Hôpitaux de Paris, Hôpital Louis Mourier, Service de Médecine Interne, Colombes, France

**Keywords:** cancer, individual patient data meta-analysis, Ottawa score, tinzaparin, venous thromboembolism

## Abstract

**Background:**

Risk of venous thromboembolism (VTE) recurrence remains high in patients with cancer-associated thrombosis (CAT), despite therapeutic anticoagulation. Identifying patients at risk of treatment failure is still a challenge.

**Objectives:**

We aimed to assess the performance of the Ottawa score in predicting VTE recurrence in a large homogeneous population of patients with CAT treated with the same anticoagulant, tinzaparin, for at least 3 months.

**Methods:**

Individual patient data from 3 prospective cohort studies and 1 randomized controlled trial were pooled (PROSPERO: CRD42019119907). Clinical events of interest were adjudicated by independent central adjudication committees in all 4 studies.

**Results:**

Among the 1413 patients included, the Ottawa score could be calculated for 1088 of whom 646 (59.4%) were classified at high risk of recurrence (Ottawa score ≥ 1). The 6-month cumulative incidence of recurrent VTE was 5.0% (95% CI, 3.2-7.8) in the Ottawa low-risk group and 8.5% (95% CI, 6.6-10.8) in the high-risk group. The area under the receiver operating characteristic curve was 0.56 (95% CI, 0.51-0.62). The sensitivity of the dichotomized Ottawa score (score ≥ 1) was 72.8% (95% CI, 62.6%-83.0%), the specificity was 41.9% (95% CI, 37.8%-45.9%), the positive predictive value was 8.6% (95% CI, 6.4%-10.8%), and the negative predictive value was 95.3% (95% CI, 93.3%-97.4%). Introducing additional predictive factors failed to significantly improve the score’s performance.

**Conclusions:**

Despite the large number of patients and anticoagulant treatment standardization, the Ottawa score failed to accurately predict recurrent VTE in patients with CAT treated with tinzaparin.

## Essentials

• Ottawa score evaluated in a pooled cohort of 1413 patients with CAT started on tinzaparin.
• Recurrent VTE rates were 5.0% in low-risk (41%) and 8.5% in high-risk (59%) groups at 6 months.
• Ottawa score poorly predicted recurrent VTE (area under the curve, 0.56).
• Adding other factors did not improve the score’s predictive accuracy.

## 1 Introduction

Compared with noncancer patients, patients with active cancer have both a 3- to 6-fold higher risk of incident venous thromboembolism (VTE) and a 3- to 6-fold higher risk of recurrent VTE despite curative anticoagulation [1]. Current guidelines for anticoagulation in cancer patients recommend use of low-molecular-weight heparin or a direct oral anticoagulant, based on randomized controlled trial results [[2], [3], [4], [5], [6], [7]]. Adequate anticoagulation treatment of cancer patients is complicated by many factors including comorbidities, polymedication, and increased risk of bleeding compared with that of noncancer patients. Ability to predict the risk of recurrent VTE during anticoagulation might facilitate selection of the best treatment option for patients with cancer-associated thrombosis (CAT) or contemplation of a more vigilant approach to care. Among the various prediction rules developed to assess the risk of recurrent VTE in patients with CAT, the most extensively studied index is the so-called Ottawa score, described by Louzada et al. [8]. Several cohort studies have attempted to validate this score with conflicting results [[9], [10], [11], [12], [13]]. Even the conclusions of meta-analyses performed on aggregated data are contradictory. One meta-analysis concluded that the original Ottawa score is an accurate tool for stratifying the risk of recurrent VTE, in particular for reliably identifying patients at high risk of recurrent VTE, but did not assess the predictive performance of the score [14], while the other showed poor performance of the score [13]. In these studies, the Ottawa score was mainly assessed in retrospective cohorts of patients with CAT included between 1995 and 2014 receiving now outdated antitumoral therapies and various types and doses of anticoagulant. Control of the variability induced by anticoagulation is essential to validate a risk score during such treatment. In a prospective cohort study of patients included from 2015 to 2016 and treated with tinzaparin, these 2 limitations were controlled, but the accuracy of the Ottawa score could not be validated [15]. This shortcoming might have been due to inability of the score to adequately capture the factors linked to increased risk of recurrent VTE. Specifically, different cancer sites (eg, lung, colorectal, breast, and hematologic) are associated with disparate VTE risks and involve the use of heterogeneous antitumor treatments varying in their thrombogenicity. In a sufficiently large population with CAT, additional risk factors could be identified permitting enhanced performance of the current score or development of an alternative score.

By analyzing individual patient data from prospective studies in cancer patients treated with a standardized tinzaparin regimen for a VTE episode, we aimed to improve the ability to predict recurrent VTE in the CAT population.

## 2 Methods

### 2.1 Protocol registration

The study protocol was prospectively registered in the International prospective register of systematic reviews (PROSPERO, https://www.crd.york.ac.uk/PROSPERO, registration number CRD42019119907). For the individual patient data meta-analysis, we adhered to the Preferred Reporting Items for Systematic Review and Meta-Analyses of Individual Participant Data [16].

### 2.2 Literature search and study identification

We sought to identify all relevant published and unpublished prospective studies, that is, observational cohorts or randomized controlled trials, including patients with CAT treated with tinzaparin after an episode of VTE and followed up for at least 6 months. An exhaustive literature search was performed without any restriction on language or publication period. The computer-assisted search was carried out on electronic databases (Medline, Web of Science, and Google Scholar), using the combination of keywords and medical subject headings terms related to treatment exposure (tinzaparin [Innohep]), outcome (eg, venous thromboembolism, deep vein thrombosis [DVT], and pulmonary embolism [PE]), and population (eg, cancer, acute venous thromboembolism, and cancer-associated thrombosis). Reference lists of retrieved articles and review were manually scanned to identify all relevant additional studies. The search was updated on December 26, 2023. The full electronic search equation is presented in Supplementary Table 1. Individual patient data were requested and obtained from the coordinating investigator of each trial included.

### 2.3 Study selection

Studies were eligible for inclusion if (1) they were observational, prospective, cohort studies or randomized controlled trials; (2) with a recruitment period after 2003 (excluding studies over 20 years old); (3) including patients with CAT treated with tinzaparin for at least 3 months and followed up for at least 6 months; (4) with individual patient data available; and (5) with adjudication of clinical events of interest (recurrent VTE and major bleeding) by an independent central adjudication committee as part of the study. For randomized controlled trials, only the tinzaparin arm was considered.

### 2.4 Data extraction

Electronic versions of the data sets were requested from the coordinating investigator in SAS format if possible, or otherwise in EXCEL or CSV format, with written details of the dictionary and coding of variables. The data required were defined and selected a priori to correspond to the minimum data required to meet the primary research objectives. The statistician collated and cleaned the data, and harmonized the databases. Additional data checks (eg, relating to data generation and harmonization, data consistency, and completeness) were performed by comparing the generation of the acquired data to published results or statistical reports. Individual patient data from each study were then pooled in a common database. The final analyses were performed on the collated and merged data set after the abovementioned steps.

Race and ethnicity data were only available in one of the included data sets [2]. In the French cohorts, collection of these data is restricted by law, and they were not recorded in the Spanish cohort. Consequently, these variables were not included in the pooled analysis.

### 2.5 End points

The primary end point of the meta-analysis was objectively confirmed recurrent VTE (DVT or PE) within the first 6 months of follow-up, either symptomatic or incidentally detected. Secondary outcomes included major bleeding and all-cause deaths during the same follow-up period. The definitions of recurrent VTE and major bleeding adjudicated by the independent central adjudication committee of each study were identical or very similar and were maintained for this meta-analysis.

### 2.6 Methodological quality of the studies

The Newcastle-Ottawa scale (NOS) was used to assess the quality of the cohort studies (or tinzaparin arms of randomized controlled studies) included in the meta-analysis [17]. The NOS is a star system containing 8 items categorized into 3 dimensions comprising (1) study selection, (2) comparability of the cohorts according to their design or analysis, and (3) study outcome. A maximum of 9 stars can be awarded for each study based on these 3 dimensions. In this meta-analysis, the second dimension of the scale concerning the comparability of cohorts was not applicable. A maximum of 7 stars could therefore be awarded for each study. Two authors independently assessed the quality of the studies included, with discrepancies between authors being resolved by discussion.

### 2.7 Statistical analysis

Individual data concerned all included patients with evaluable data for recurrent VTE. The Ottawa score was calculated by adding or subtracting the stated number of points for the following baseline data: female gender (+1 point), TNM stage I (−2 points), lung cancer (+1 point), breast cancer (−1 point), and a history of previous VTE (+1 point) [8]. Patients with a score of <1 were classified as being at low risk of recurrence, and those with a score of ≥1 as being at high risk of recurrence. To assess overall discrimination, the area under the receiver operating characteristic (ROC) curve of the Ottawa score was calculated with its 95% CI. The performance of the dichotomized Ottawa score (score ≥ 1) was assessed according to its sensitivity, specificity, and positive and negative predictive values. To screen for additional potential risk factors for recurrent VTE up to 6 months, the Fine and Gray regression model, considering death as a potential competing risk, was used, adjusted on the variables of the Ottawa score. This model generated subdistribution hazard ratios (subHR) with their corresponding 95% CI. The following additional variables were also tested: age, Eastern Cooperative Oncology Group index, type of tumor, evolutionary stage, renal function, and anticancer therapies. Univariate analyses were first implemented for each of these factors. Factors with a *P* value <.15 and a frequency > 3% were then introduced into a multivariate model adjusted on the variables of the Ottawa score. Variables significant at a threshold of 5% were considered for construction of the risk score for recurrent VTE. A multivariate model, including the variables significant at a threshold of 5% in the step-by-step model, was then constructed, and its Wolbers c-statistic was calculated [18]. An enhanced score was subsequently developed by adding the points assigned to each significant variable based on the regression coefficients calculated in the multivariate model. The predictive power of this score was evaluated in the study cohorts, and the area under the ROC curve associated with the score was determined. This newly constructed enhanced score was internally validated, with its predictive power being evaluated by the bootstrapping method. The same methods were used to develop an alternative score designed to improve the prediction of recurrence risk. All analyses were performed using SAS software (version 9.4; SAS Institute). R software (version 4.2.1; R Core Team) was used to generate graphs.

## 3 Results

### 3.1 Study selection

**Figure 1:**
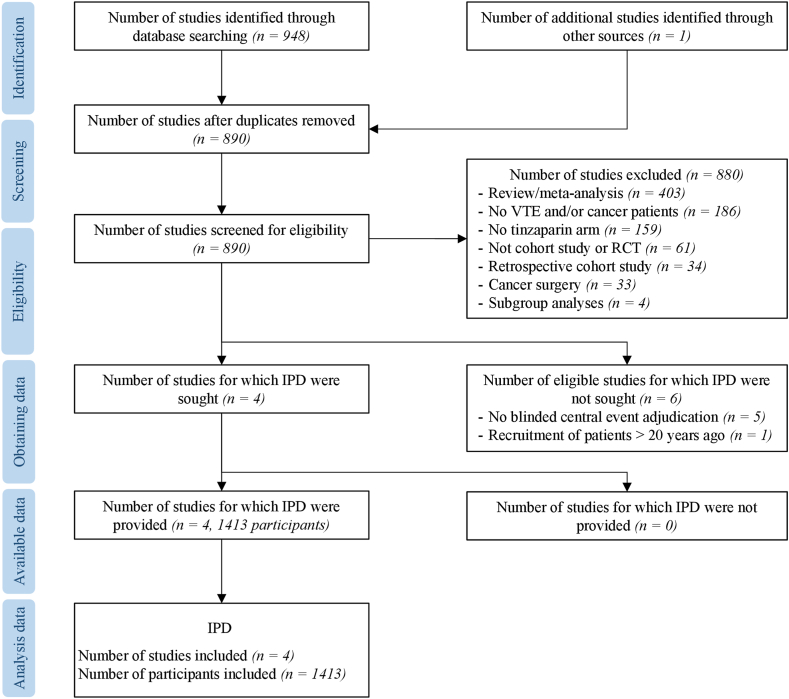
Flow chart. IPD, individual patient data; RCT, randomized controlled trial; VTE, venous thromboembolism.

The search procedure identified 949 references (Figure 1). After exclusion of 939 studies, including 59 duplicates, 10 studies were potentially eligible for analysis. For 6 of these studies, individual patient data were not sought owing to the lack of blinded central event adjudication [13,[19], [20], [21], [22]] and the fact that the patients were recruited >20 years ago [23]. Finally, individual patient data were available for 3 prospective cohort studies—TiCAT [24], PREDICARE [15], and AXA (not yet published) and 1 randomized controlled study (CATCH [2]) (Table 1). All these studies were multicenter. Two studies were sponsored by LEO Pharma [2,15], and the others by University Hospitals in France (AXA) and Spain (TiCAT) [24]. In total, 1413 patients were treated with tinzaparin, with the number of treated patients per study varying from 247 [24] to 449 [2]. All studies attained the maximum of 7 stars on the NOS.

**Table 1:** Design of studies included in the meta-analysis.

Study	TiCAT [24]	CATCH [2]	PREDICARE [15]	AXA (not yet published)
Country	Spain	International	France	France
Sponsor	Hospital Universitario Virgen del Rocío	LEO Pharma (NCT01130025)	LEO Pharma (NCT03099031)	AP-HP (NCT02898051)
Inclusion period	January 2009 to September 2015	August 2010 to November 2013	May 2015 to April 2016	August 2011 to November 2015
Design	Prospective cohort study of tinzaparin-treated patients	Tinzaparin arm of an open RCT	Prospective cohort study of tinzaparin-treated patients	Prospective cohort study of tinzaparin-treated patients
No. of centers	3	164	75	8
No. of patients	247	449	409	308

AP-HP, Assistance Publique – Hôpitaux de Paris; RCT, randomized controlled trial.

### 3.2 Patients

Baseline patient characteristics and the 6-month cumulative incidences of recurrent VTE, major bleeding, and death from any cause are presented by individual study and for the overall study population in Table 2. The median age of the patients was 65.0 years (IQR, 56.0-73.5 years), and 51.9% were females. Regarding fragilities, 21.3% of patients were aged ≥75 years, 9.3% had a body weight of ≤50 kg, and 13.9% had a creatinine clearance <50 mL/min. The qualifying VTE event was symptomatic in 75.0% of patients, 23.0% presenting both DVT and PE. The primary tumor site was gastrointestinal in 27.0% of patients, pulmonary in 18.0%, genitourinary in 14.1%, gynecologic in 14.0%, breast in 11.7%, hematologic in 7.4%, and elsewhere in 7.8%. The cancer was metastatic in 61.7% of patients.

**Table 2:** Baseline characteristics of patients with CAT enrolled in each study and in the overall meta-analysis population.

Characteristic	TiCAT [24] (*n* = 247)	CATCH [2] (*n* = 449)	PREDICARE [15] (*n* = 409)	AXA (*n* = 308)	Total (*n* = 1413)
Age (y), median (IQR)	64.8 (56.0-72.5)	60.0 (52.0-69.0)	68.3 (59.4-76.7)	67.5 (59.9-75.7)	65.0 (56.0-73.5)
Age ≥ 75 y	42 (17.1)	50 (11.1)	123 (30.1)	86 (27.9)	301 (21.3)
Female gender	112 (45.3)	262 (58.4)	199 (48.7)	160 (51.9)	733 (51.9)
BMI (kg/m^2^), median (IQR)	NA	24.8 (21.7-28.6)	24.5 (21.7-28.0)	24.9 (22.0-28.1)	24.7 (21.9-28.3)
Body weight ≤ 50 kg	4 (1.9)	67 (14.9)	33 (8.1)	25 (8.1)	129 (9.3)
Creatinine clearance < 50 mL/min	17 (7.8)	66 (14.9)	57 (14.5)	49 (15.9)	189 (13.9)
History of previous VTE	NA	27 (6.0)	49 (12.0)	61 (19.8)	137 (11.7)
Qualifying thrombotic event					
DVT only	128 (51.8)	252 (57.1)	162 (39.6)	64 (20.8)	606 (43.2)
PE only	75 (30.4)	48 (10.9)	175 (42.8)	177 (57.7)	475 (33.8)
DVT and PE	44 (17.8)	141 (32.0)	72 (17.6)	66 (21.5)	323 (23.0)
Symptomatic event	168 (68.0)	441 (100.0)	271 (66.3)	174 (56.5)	1054 (75.0)
ECOG 0-1	206 (85.1)	343 (76.4)	239 (58.7)	146 (60.8)	934 (69.8)
Primary tumor site					
Gastrointestinal^a^	45 (18.2)	125 (27.8)	108 (26.4)	104 (33.8)	382 (27.0)
Upper	6 (2.4)	29 (6.5)	15 (3.7)	13 (4.2)	63 (4.5)
Lower	29 (11.7)	66 (14.7)	59 (14.4)	65 (21.1)	219 (15.5)
Digestive tract	10 (4.0)	30 (6.7)	34 (8.3)	26 (8.4)	100 (7.1)
Lung	41 (16.6)	48 (10.7)	128 (31.3)	37 (12.0)	254 (18.0)
Genitourinary^b^	53 (21.5)	53 (11.8)	42 (10.3)	51 (16.6)	199 (14.1)
Gynecologic^c^	22 (8.9)	101 (22.5)	33 (8.1)	42 (13.6)	198 (14.0)
Breast	35 (14.2)	37 (8.2)	57 (13.9)	37 (12.0)	166 (11.7)
Hematological	20 (8.1)	44 (9.8)	22 (5.4)	18 (5.8)	104 (7.4)
Other sites	31 (12.6)	41 (9.1)	19 (4.6)	19 (6.2)	110 (7.8)
Metastatic cancer	161 (65.7)	247 (55.0)	268 (67.0)	179 (61.3)	855 (61.7)
Cancer treatment					
Chemotherapy	154 (66.1)	133 (29.6)	263 (64.3)	199 (64.8)	749 (53.6)
Radiotherapy	11 (7.1)	51 (11.4)	48 (11.7)	21 (6.8)	131 (9.9)
Recurrent VTE	11	31	28	15	85
Cumulative incidence (%) (95% CI)^d^	4.6 (2.6-8.0)	7.2 (5.0-10.3)	7.3 (5.3-10.2)	5.0 (2.9-8.3)	6.2 (5.0-7.7)
Major bleeding	7	13	15	12	47
Cumulative incidence (%) (95% CI)^d^	3.0 (1.5-6.0)	3.0 (1.6-5.7)	3.7 (2.3-6.1)	3.9 (2.2-7.0)	3.4 (2.7-4.5)
All-cause deaths	38	156	144	88	426
Cumulative incidence (%) (95% CI)^e^	16.2 (11.5-20.9)	36.4 (31.8-41.0)	36.2 (31.3-41.0)	29.4 (24.2-34.6)	31.3 (28.9-33.8)

Values are *n* (%) unless stated otherwise.

BMI, body mass index; ECOG, Eastern Cooperative Oncology Group; NA, not available; VTE, venous thromboembolism.

a Colorectal, pancreas, stomach, biliary tract, esophagus, small intestine, peritoneum, jejunum, and ampulla of Vater.

b Prostate, kidney, bladder, testicular, penile, and ureter.

c Ovary, cervix, uterus, endometrium, vagina, vulva, fallopian tube, and female reproductive system.

d Estimated by the Kalbfleisch and Prentice method taking into account the competing risk of death.

e Estimated by the Kaplan-Meier method.

The 6-month cumulative incidence of recurrent VTE varied from 4.6% in TiCAT to 7.3% in PREDICARE (Supplementary Figure 1A). Overall, recurrent VTE occurred in 85 patients, yielding a 6-month cumulative incidence of recurrent VTE of 6.2% (95% CI, 5.0-7.7). Regarding major bleeding, the 6-month cumulative incidence of major bleeding varied from 3.0% in the TiCAT and CATCH studies to 3.9% in the AXA study (Supplementary Figure 1B). Overall, major bleeding occurred in 47 patients, yielding a 6-month cumulative incidence of 3.4% (95% CI, 2.7-4.5). A total of 426 patients died, mainly from the underlying cancer (31.3%; 95% CI, 28.9-33.8) (Supplementary Figure 1C).

### 3.3 Performance of the Ottawa score

**Figure 2:**
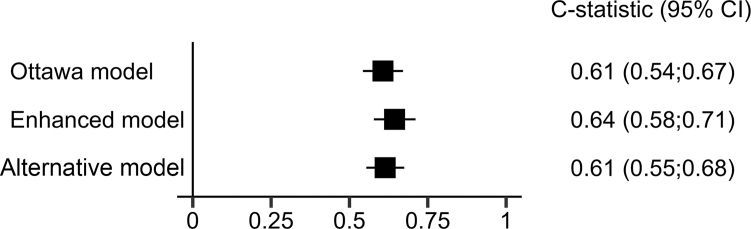
Wolbers c-statistic associated with the 3 models.

Data for the variables in the multivariate model used to calculate the Ottawa score were available for 1088 of the 1413 patients included, as data on tumor stage and a personal history of VTE were not available in the TiCAT study. The c-statistic associated with this model was 0.61 (95% CI, 0.54-0.67) (Figure 2).

**Figure 3:**
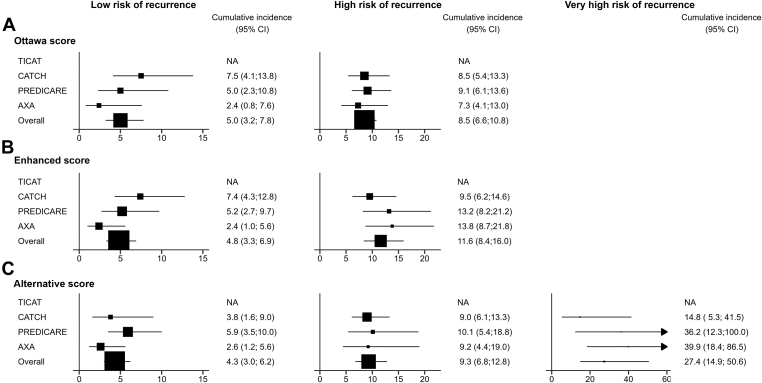
Cumulative incidence of recurrent venous thromboembolism according to the risk scores in each study and in the overall meta-analysis population.

The Ottawa score classified 442 patients (40.6%) as at low risk of recurrence (Ottawa score < 1), with an estimated 6-month cumulative recurrent VTE incidence of 5.0% (95% CI, 3.2-7.8), and 646 patients (59.4%) as at high risk of recurrence with a 6-month cumulative recurrent VTE incidence of 8.5% (95% CI, 6.6-10.8) (Figure 3). The area under the ROC curve was 0.56 (95% CI, 0.51-0.62). The sensitivity, specificity, and negative and positive predictive values were 72.8%, 41.9%, 8.6%, and 95.3%, respectively (Table 3).

**Table 3:** Performance of the Ottawa score, enhanced score, and alternative score.

Score category	Ottawa score (score range, −3 to 3)	Enhanced score (score range, −4 to 5)	Alternative score (score range, −3 to 2)
Low risk of recurrence (score)			
Cutoff value	<1	≤1	≤0
No. of patients	442	673	665
Cumulative incidence (%) (95% CI)^a^	5.0 (3.2-7.8)	4.8 (3.3-6.9)	4.3 (3.0-6.2)
High risk of recurrence (score)			
Cutoff value	≥1	≥2	1
No. of patients	646	375	430
Cumulative incidence (%) (95% CI)^a^	8.5 (6.6-10.8)	11.6 (8.4-16.0)	9.3 (6.8-12.8)
Very high risk of recurrence (score)			
Cutoff value	—	—	2
No. of patients	—	—	25
Cumulative incidence (%) (95% CI)^a^	—	—	27.4 (14.9-50.6)
Performance of the score			
AUC (95% CI)	0.56 (0.51-0.62)	0.59 (0.53-0.65)	0.59 (0.52-0.65)
Sensitivity (95% CI)	72.8 (62.6-83.0)	59.1 (47.8-70.4)	9.6 (2.8-16.4)
Specificity (95% CI)	41.9 (37.8-45.9)	61.2 (57.2-65.2)	97.7 (96.5-98.9)
Positive predictive value (95% CI)	8.6 (6.4-10.8)	10.3 (7.3-13.3)	22.7 (6.8-38.7)
Negative predictive value (95% CI)	95.3 (93.3-97.4)	95.2 (93.5-96.9)	93.9 (92.4-95.3)

AUC, area under the curve.

a Estimated by the Kalbfleisch and Prentice method to take into account the competing risk of death.

### 3.4 Attempts to enhance the Ottawa score

In a multivariate analysis adjusted for the variables included in the Ottawa score (Table 4), the following additional factors were independently associated with the risk of recurrent VTE: proximal DVT at inclusion (subHR, 2.01; 95% CI, 1.18-3.43), surgery or trauma in the previous month (subHR, 2.51; 95% CI, 1.22-5.14), and colorectal cancer (subHR, 0.34; 95% CI, 0.13-0.85). Bootstrapping analysis confirmed these results. The c-statistic of this enhanced model adjusted for the variables included in the Ottawa score was 0.64 (95% CI, 0.58-0.71) (Figure 2).

**Table 4:** Results of the enhanced analysis.

Variables included in the enhanced model	Regression coefficients (SD)	subHR (95% CI)	*P*	Scoring
Female gender^a^	—	—	—	+1
Tumor stage I^a^	—	—	—	−2
Lung cancer^a^	—	—	—	+1
Breast cancer^a^	—	—	—	−1
Personal history of VTE^a^	—	—	—	+1
Proximal DVT at inclusion	0.70 (0.27)	2.01 (1.18-3.43)	.01	+1
Surgery or trauma (in the previous month)	0.92 (0.37)	2.51 (1.22-5.14)	.01	+1
Colorectal cancer	−1.09 (0.47)	0.34 (0.13-0.85)	.02	−1

DVT, deep vein thrombosis; subHR, subdistribution hazard ratio; VTE, venous thromboembolism.

a Variables included in the Ottawa score.

The score developed from this model ranged from -4 to 5 points and was dichotomized as follows: patients with a score of ≤1 were considered at low to moderate risk of VTE recurrence, and patients with a score of ≥2 were considered at high to very high risk of VTE recurrence. The 6-month cumulative incidence of recurrent VTE was 4.8% (95% CI, 3.3-6.9) in the low- to moderate-risk group and 11.6% (95% CI, 8.4-16.0) in the high- to very high–risk group (Figure 3). The predictive performance of this enhanced score was no better than that of the original Ottawa score (Table 3).

### 3.5 Attempts to develop an alternative predictive model

It was assumed that data on patients from all 4 studies could contribute to the development of an alternative predictive model. In the multivariate analysis (Table 5), the following factors were independently associated with the risk of recurrent VTE: surgery or trauma during the previous month (subHR, 2.55; 95% CI, 1.27-5.14), proximal DVT at inclusion (subHR, 1.90; 95% CI, 1.13-3.20), lower gastrointestinal cancer (mainly colorectal cancer; subHR, 0.36; 95% CI, 0.15-0.83), and breast cancer (subHR, 0.10; 95% CI, 0.01-0.72). Bootstrapping analysis confirmed these results. The c-statistic of this model was 0.61 (95% CI, 0.55-0.68) (Figure 2).

**Table 5:** Results of the alternative predictive model.

Variables included in the alternative model	Regression coefficient (SD)	subHR (95% CI)	*P*	Scoring
Proximal DVT at inclusion	0.64 (0.27)	1.90 (1.13-3.20)	.02	+1
Surgery or trauma (in the previous month)	0.94 (0.35)	2.55 (1.27-5.14)	.009	+1
Breast cancer	−2.30 (1.01)	0.10 (0.01-0.72)	.02	−2
Lower GI cancer^a^	−1.03 (0.43)	0.36 (0.15-0.83)	.02	−1

DVT, deep vein thrombosis; subHR, subdistribution hazard ratio.

a Excluding esophagus, stomach, and pancreas.

The alternative score developed from this model ranged from −3 to 2 points and comprised 3 categories, as follows: patients with a score of ≤0 were considered at low to moderate risk of recurrence of VTE, those with a score of 1 were considered at high risk of recurrence of VTE, and those with a score of 2 were considered at very high risk of recurrence of VTE. The 6-month cumulative incidence of recurrent VTE was 4.3% (95% CI, 3.0-6.2) in the low- to moderate-risk group, 9.3% (95% CI, 6.8-12.8) in the high-risk group, and 27.4% (95% CI, 14.9-50.6) in the very high–risk group (Figure 3). The predictive performance of this alternative score was no better than that of the original Ottawa score (Table 3).

## 4 Discussion

In this individual patient–data meta-analysis including 1413 patients with CAT treated with tinzaparin, the Ottawa score failed to accurately predict recurrent VTE. Although this score was able to stratify patients by risk of recurrent VTE, showing a lower cumulative incidence in low-risk patients—that is, with an Ottawa score of <1—than that in high-risk patients (5.0% vs 8.5%), its overall predictive performance was poor, with an area under the ROC curve of only 0.56. This study also failed to identify alternative significant clinical factors that could help better predict the risk of recurrent VTE in patients with CAT.

After this study was registered in PROSPERO, 2 meta-analyses aiming to validate the Ottawa score were published. An unregistered meta-analysis involving patients with CAT treated with various low-molecular-weight heparins failed to validate the Ottawa score [13]. The second meta-analysis concluded that the original Ottawa score effectively stratified the risk of recurrent VTE, concurring with findings from the present meta-analysis [14]. However, neither meta-analysis assessed the predictive performance of this score. Importantly, substantial heterogeneity was observed both between and within the individual studies incorporated, with regard to inclusion criteria, types of anticoagulant and anticancer treatments, study design, and outcomes. The performance of the Ottawa score in a more homogeneous and contemporary patient population therefore remained unexplored, justifying the present analysis. This meta-analysis, focusing exclusively on prospective studies using a standardized tinzaparin regimen and independent central outcome adjudication, aimed to address the key limitations of previous validation studies, particularly the heterogeneity in treatment protocols and diagnostic criteria. However, even under these more controlled and homogeneous conditions, the Ottawa score demonstrated only modest discriminatory ability. This suggests that its limited performance is not merely attributable to methodological variability but rather reflects an intrinsic limitation of the score itself in reliably identifying patients truly at high risk of recurrence.

Nevertheless, the negative predictive value in our cohort was 95.3%, indicating that patients classified as low risk (Ottawa score < 1) had a very low likelihood of recurrent VTE within the first 6 months. Although the sensitivity and specificity of the score were limited, its high negative predictive value, partly due to the relatively low (<10%) incidence of recurrent VTE even among high-risk patients, may still provide reassurance in identifying patients at lower risk of recurrence while on anticoagulation. From a clinical perspective, the Ottawa score is not intended to guide the discontinuation of anticoagulation, which remains recommended for all patients with CAT for at least 6 months. Nevertheless, it may still provide useful information for risk assessment and follow-up strategies during ongoing therapy.

To enhance the predictive accuracy of this score, we tested additional clinical variables associated with the risk of thrombosis. Although factors such as recent surgery or trauma and proximal DVT increased the risk of VTE recurrence, whereas colorectal or breast cancer were linked to a lower risk, integrating these factors into the enhanced and alternative scores did not substantially improve performance (c-statistics < 0.65). The alternative model stratified risk modestly, identifying a very high–risk group, but its overall clinical utility remains limited. Notably, recent surgery or trauma was independently associated with recurrence, challenging the common view of surgery as a short-term risk factor. In cancer patients, surgery may lead to prolonged prothrombotic conditions due to factors such as extended immobility, central venous access, treatment delays, and systemic inflammation, possibly justifying longer anticoagulation in this population [25,26].

These findings highlight the complexity of predicting recurrent VTE in cancer patients. The heterogeneity of cancer types, evolving anticancer treatments, and dynamic clinical states likely limit the utility of static baseline risk scores. Our results support the need for individualized assessments rather than reliance on current predictive tools. Future models may benefit from incorporating dynamic or time-varying variables, such as treatment response, biomarkers, or imaging findings, possibly using machine learning techniques on large prospective data sets to account for complex interactions. Pending the development of more accurate tools, caution is warranted in using the Ottawa score to guide management decisions in patients with CAT.

Our study has several strengths, including a large, homogeneous population of prospectively recruited patients, rendered possible by effective data sharing and collaborations. The inclusion of all eligible studies underscores the invaluable contribution of data sharing in contemporary clinical research. However, several limitations should be noted. First, data collection was not standardized across the different cohorts. Variability in definitions, data quality, and completeness may have introduced misclassification or residual confounding, particularly for key clinical variables. Notably, race and ethnicity data were available in only 1 of the included data sets and could not be included in the pooled analyses. This heterogeneity, despite harmonization efforts, may have impacted the reliability of predictive modelling. Second, the score itself has limitations: while it is applicable to breast and lung cancer, it does not apply to the full spectrum of tumor sites, despite the known differences in the risk of recurrent VTE by cancer type [27]. Third, only 3 studies contributed to validation of the enhanced and alternative scores as data on recent surgery or trauma were not collected in the TiCAT study. However, this was not necessarily a power issue, as increased power would increase precision rather than performance. Importantly, TiCAT patients contributed substantially to the analysis of other predictors of recurrence. Finally, although the original literature search was conducted in December 2023, an updated search covering the period from December 2023 to October 2025 did not yield any additional eligible studies.

## 5 Conclusion

Despite the use of individual data from 4 large, recent, prospective cohorts of patients with CAT treated with tinzaparin, this meta-analysis failed to demonstrate the clinical relevance of the Ottawa score in predicting VTE recurrence over the first 6 months of anticoagulant therapy. As high VTE recurrence rates persist despite anticoagulation in patients with CAT, further research is needed to identify more accurate and personalized predictors of recurrence, ideally in even more homogeneous patient populations, taking into account cancer type and treatment.
