# Supplementary material for: The Ottawa score for prediction of recurrent venous thromboembolism in cancer patients treated with tinzaparin: an individual patient data meta-analysis
Source: Res Pract Thromb Haemost. 2025 Dec 2;10(1):103278. doi: 10.1016/j.rpth.2025.103278 (PMC12856147; doi:10.1016/j.rpth.2025.103278)
Supplement: Supplementary Material [file mmc1.docx]

# The Ottawa score for prediction of recurrent venous thromboembolism in cancer patients treated with tinzaparin: an individual patient data meta-analysis

# Appendices

**Fig. A. 1.** Cumulative incidence of recurrent venous thromboembolism, major bleeding and all-cause deaths at 6 months in each study and in the overall meta-analysis population.


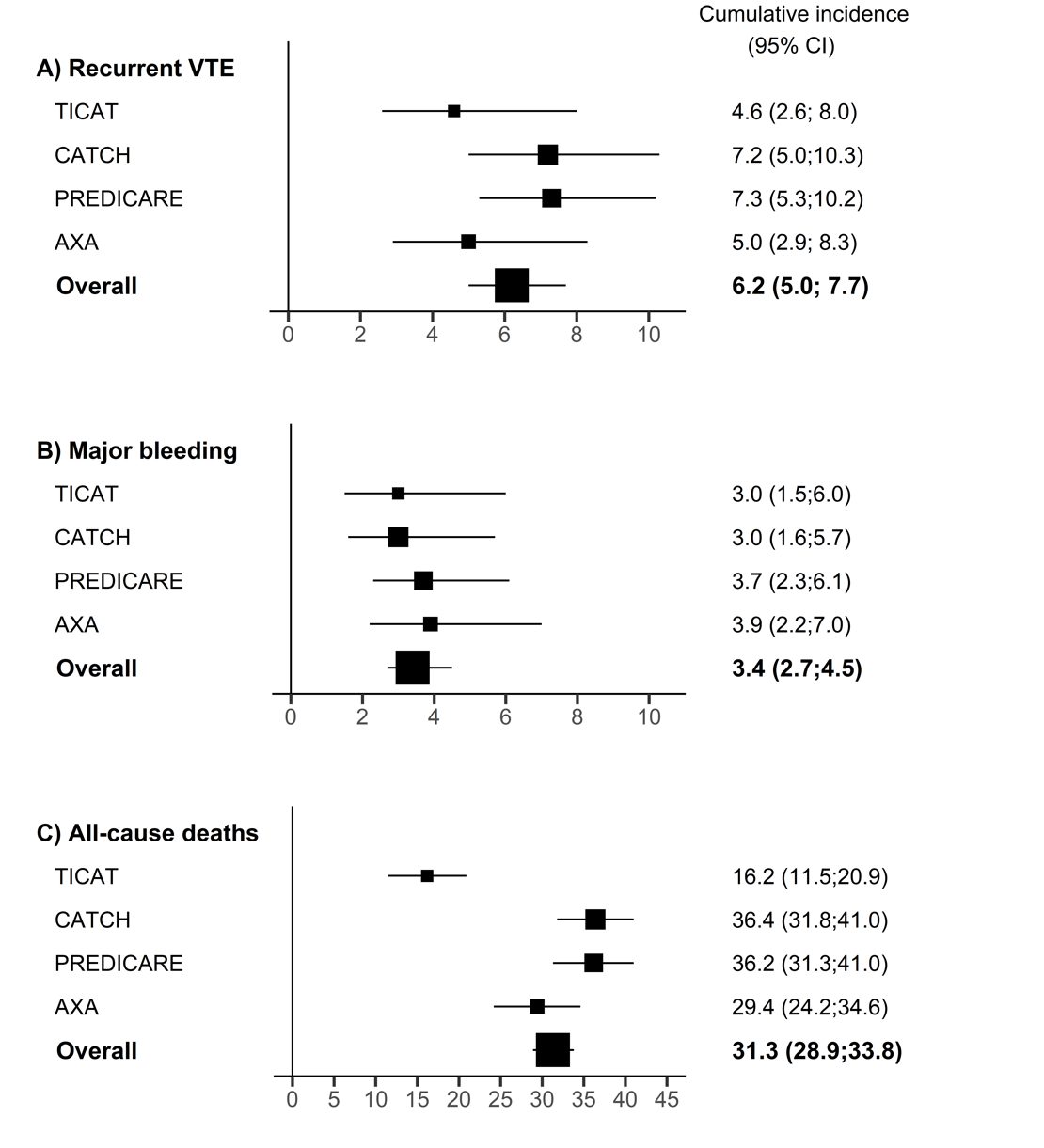


95% CI: 95% confidence interval

**Table A. 1.** Full electronic search equations used for studies identification electronic databases up to December 26^th^, 2023

| **Sources** | **Search strategies** |
| --- | --- |
| Pubmed |  |
| **1** | **((“pulmonary embolism”[MeSH Terms]) OR (“venous thromboembolism”[MeSH Terms]) OR (“venous thrombosis”[MeSH Terms]) OR (“venous thromboembolism/drug therapy”[MeSH Terms]) OR (“Pulmonary Embolism/drug therapy”[MeSH Terms]) OR (“thrombosis”[Title]) OR (“venous thrombosis”[Title]) OR (“venous thromboembolism*”[Title]) OR (“venous thrombo-embolism”[Title]) OR (“VTE”[Title]) OR (“**Thrombosis/drug therapy”**[MeSH Terms])** OR **(“deep venous thrombosis”[Title]) OR (“deep vein thrombosis”[Title]))** |
| **2** | (("heparin, low molecular weight/administration and dosage"[MeSH Terms]) OR ("heparin, low molecular weight/adverse effects"[MeSH Terms]) OR ("heparin, low molecular weight/pharmacology"[MeSH Terms]) OR ("heparin, low molecular weight/therapeutic use"[MeSH Terms]) OR (“LMWH”[Title/Abstract]) OR (“low molecular weight heparin”[Title/Abstract]) OR (“low-molecular-weight-heparin”[Title/Abstract]) OR ("tinzaparin/administration and dosage"[MeSH Terms]) OR ("tinzaparin/adverse effects"[MeSH Terms]) OR ("tinzaparin/pharmacology"[MeSH Terms]) OR ("tinzaparin/therapeutic use"[MeSH Terms]) OR ("tinzaparin"[Title/Abstract]) OR ("innohep"[Title/Abstract])) |
| **3** | ((“cancer*”[Title]) OR (“malignancy”[Title/Abstract]) OR (“malignancies”[Title/Abstract]) OR (“oncology patients”[Title/Abstract]) OR (“cancer associated thrombosis”[Title/Abstract]) OR (“cancer-associated thrombosis”[Title/Abstract]) OR (“CAT patients”[Title/Abstract]) OR (“Neoplasms/pathology”[MeSH Terms]) OR (“Neoplasms/drug therapy”[MeSH Terms])) |
| **4** | (("randomized controlled trials as topic"[MeSH Terms]) OR ("randomized controlled trial"[Publication Type]) OR ("controlled clinical trials as topic"[MeSH Terms]) OR ("randomised"[Title/Abstract]) OR ("randomized"[Title/Abstract]) OR ("prospective studies"[MeSH Terms]) OR ("cohort studies"[MeSH Terms]) OR ("prospective cohort study"[Title/Abstract]) OR ("prospective study"[Title/Abstract]) OR (("cohort study"[Title/Abstract]) OR ("prospective"[Title/Abstract]))) |
| **5** | (“recommendations”[Title] OR “guidance”[Title] OR “guidelines”[Title]) |
| **6** | (“design”[Title] OR “protocol”[Title]) |
| **7** | “adherence”[Title] |
| **8** | (“surgery”[Title] OR “surgeries”[Title]) |
| **9** | (“knee”[Title] OR “hip”[Title] OR “arthroplasty”[Title] OR “arthroplasties”[Title]) |
| **10** | (“secondary prevention”[Title] OR “extended”[Title]) |
| **11** | (“post-thrombotic syndrome”[Title] OR “postthrombotic syndrome”[Title]) |
| **12** | **1 AND 2 AND 3 AND 4 NOT 5 NOT 6 NOT 7 NOT 8 NOT 9 NOT 10 NOT 11** |
| Web of science | (venous thromboembolism OR venous thrombosis OR deep venous thrombosis OR deep vein thrombosis OR DVT OR pulmonary embolism OR PE) (Topic) AND (tinzaparin OR innohep) (Topic) AND (cancer OR malignancy OR malignancies OR cancer associated thrombosis OR cancer-associated thrombosis OR CAT patients OR neoplasms) (Topic) |
| Google scholar | (venous thromboembolism OR venous thrombosis OR deep venous thrombosis OR deep vein thrombosis OR DVT OR pulmonary embolism OR PE) AND (cancer OR malignancy OR malignancies OR cancer associated thrombosis OR cancer-associated thrombosis OR CAT patients OR neoplasms) AND (tinzaparin OR innohep) -guidelines -recommandations -guidance -design -protocol -adherence -surgery -surgeries -knee -hip -arthroplasty -arthroplasties -extended -post-thrombotic -review -meta-analysis -thromboprophylaxis -prevention -extended |
| Clinicaltrials.gov | (venous thromboembolism OR venous thrombosis OR deep venous thrombosis OR deep vein thrombosis OR DVT OR pulmonary embolism OR PE) AND (cancer OR malignancy OR malignancies OR cancer associated thrombosis OR cancer-associated thrombosis OR CAT patients OR neoplasms) AND (tinzaparin OR innohep) |
